# Removal of Solid Ovarian Tumor, with Grave Complications

**Published:** 1891-05

**Authors:** J. McFadden Gaston

**Affiliations:** Atlanta, Ga.


Southern Medical Record.

A MONHLY JOURNAL OF MEDICINE AND SURGERY.

VOL. XXI. ATLANTA, GA., MAY, 1891. NO. 5.

### Original Articles

### REMOVAL OF SOLID OVARIAN TUMOR, WITH GRAVE COMPLICATIONS.

BY J. MCFADDEN GASTON, M. D., ATLANTA, GA.

Read before the Medical Association of Georgia, at Augusta, April 16. 1891.

The following communications from Dr. James J. Winn, of Clayton, Alabama, in regard to the patient's previous history, were received at the dates given:

"January 27th, 1891. A few weeks since I was called to see a white woman, married, 45 years old, mother of six or eight children. Found her vital forces almost nil in consequence of extreme pressure from abdominal tumor, which developed since July last. I used aspiration, removing a large quantity (7 qts.) of heavy straw-colored fluid. Saw her again three weeks subsequently—general condition very much improved, but abdomen rapidly refilling. Aspirated again, removing one gallon. Solid portion of tumor has very much increased. The tumor is, I think, ovarian."

"February 18th. I saw Mrs. H. yesterday, and directed her to take train for your city to-morrow. She has had a right severe attack of phlebitis, which leaves her lower limbs decidedly swollen. However, as her bowels, digestion, etc., were in pretty good condition, I decided to send her at once. Hope you will not find the case complicated pregnancy.

"If you decide to operate, write me particulars and character of tumor."

The patient referred to in the above communication arrived in Atlanta on the night of February 19th, and was taken to the Providence Infirmary.

She was examined next morning, and found in a very feeble condition from the fatigue of travel, added to her previous prostration. As soon as alcohol stimulants had been used without relief to the nausea and retching, it was thought proper to give her calomel, grain 1-2, bicarb. soda, grains iii., at intervals of three hours until she took three doses. This afforded marked relief, and was followed by tablespoonful doses every two or three hours of lime water 3 ounces, camphor water 2 ounces, and peppermint water 1 ounce.

There was considerable accumulation of fluid in the abdomen, with evidence of solid mass in the pelvic region extending nearly up to the umbilicus and occupying the space on either side to the illa, with projection downward into Douglas' cul de sac.

The left lower extremity was greatly enlarged, assuming the characteristics of phlegmasia dolens. This was smeared from the foot up to the body with the following:

Mercurial ointment, belladonna ointment, each 1 ounce; pulv. camphor, 1 drachm, thoroughly mixed. Outside of this, cotton batting was secured by a roller, applied firmly, so as to keep up compression.

February 21st. The stomach being improved, she took sul. quin., grains 24, bicarb. soda, grains 60, in 12 capsules, one every three hours.

In the next two days the limb had become so much smaller as to necessitate the re-application of the bandage. But the abdominal enlargement had increased.

Februry 24th. As a tonic and alterative, the following was prescribed:

| | | |
|---|---|---|
| ℞. | Huxam's tincture, . . . | f℥ii. |
| | Tinct. nux vomica, . . . | fʒi. |
| | Chlorate potash, . . . | ʒi. |
| | Water, q. s. . . . . . . | f℥vi. |

Mix and take a tablespoonful every four hours.

While her general health improved under this medicine, and the left lower extremity was reduced to nearly its natural size, there was discovered an anasarous condition of the right limb, with marked increase of the abdomen. The right limb was treated as the other had been, and she took internally the following, commencing March 1st:

R. Calomel, . . . . grs. vi.
Pulv. digit., . . . grs. xii.
Pulv. squill, . . . grs. xviii.

Mix in 12 capsules. Take one every four hours.

This combination acted upon the bowels so as to require the intervals between the doses to be lengthened to six hours, and was attended with relief to the dropsical effusion. But notable depression of the vital forces followed, calling for the use of diffusible stimulants, such as Hoffman's anodyne, Ar. spts. ammonia, etc.

The swelling in the left lower extremity having almost disappeared, and the anasarcous condition of the right having diminished, while the abdominal distension had materially increased, I concluded that an operation was indicated as the choice of evils.

Accordingly, on March 10th, 1891, with the co-operation of Dr. J. G. Earnest, and the assistance of Drs. Elkin, Roy, Boland and Renouff, the laparotomy was performed under the influence of the A. C. E. mixture.

As the material facts are given in my reports to Dr. Winn, they may be here recorded with the correspondence:

March 10th, 1891. The laparotomy in the case of Mrs. H. was performed to-day at 12 o'clock, and now, five hours afterwards, she has recovered from the effects of the anæsthetic, but still manifesting great depression of vital forces (shock). Hypodermics of whisky were used before completing the operation, and have been kept up since, with the use of morphine, grain 1-4, atropia, 1-150 grain about an hour ago.

The adhesions were extensive, and the oozing from the surfaces caused some loss of blood, while the pedicle was very broad and encroaching upon the left corner of the uterus, so that the ligation was attended with difficulty. The fluid was in the peritoneal cavity, independent altogether of the solid tumor.

It will be preserved, so that you may see it if you should favor us with a visit at some future day.

And yet, nothing definite can be said as to the recovery of the patient, and I am seriously apprehensive that she cannot rally from the shock. Should her powers hold out for 24 hours, there would be a reasonable ground to expect a favorable result.

March 11th, 1891. I wrote yesterday, giving the result of the operation on Mrs. H., and giving rather a gloomy outlook. She has rallied from the shock, and at 10 o'clock a. m. I find her comparatively free from risk on that score ; but the prostration of her forces prior to the operation, with hydropic tendency, should predispose her to typhoid condition, even if she escapes peritonitis. I am therefore still in doubt as to the prognosis, but hopeful. She has taken by enema whisky with bovanine during the night, and quinine is added to these to-day. A little milk punch has been taken by the mouth.

March 12th. There is no great change in the condition of our patient, yet the fact of holding up another day argues favorably. Her temperature is only 99 1-4 degress, and pulse 130 per minute, without any special sensations over the abdomen. She is disposed to look forward to recovery, and is rather cheerful, but without much physical strength, so that no definite calculation can be made as to the result.

March 13th. At the morning visit (11 a. m.) to-day, to our patient, she is found with pulse and temperature corresponding to report of yesterday, and some improvement in strength. Having occasion to change the dressings (which were soiled by involuntary discharge of urine), the line of incision was apparently closed by adhesive inflammation, and there was no tympanitis; but percussion over the abdomen revealed moderate sensitiveness. She is in good spirits, and having passed three days now without anything untoward, I trust all may go well with her.

March 14th. There are some changes to be noted in our patient's condition to-day, the temperature being abnormal, 97 3-4, pulse 120 per minute, with tendency to vomiting and general prostration. The sensitiveness over the abdomen has not materially increased, and there is no tympanitis.

She has been taking 5 grains of quinine night and morning, with morphia, grain 1-4, atropia, grain 1-150, when restless, not more than twice in 24 hours. I directed, at 11 a. m., calomel, grain 1-2, with bicarb. soda, grains 3, every four hours, and that she should have champagne with ice frequently. She used the milk punch, and this was to be substituted with milk and lime water. The adynamic condition causes me to apprehend septicæmia.

March 15th. The indications of threatened septicæmia which I reported yesterday have not been confirmed by any aggravation of our patient's condition, though her temperature continues at 97 3-4 degrees, with a pulse 116 beats. The state of her stomach precludes taking much nourishment, and she could not retain the iced champagne. Three doses of cal., grain 1-2, bicarb. soda, grains 3, led to frequent fœcal evacuations without causing any considerable diminution of strength. I directed a return to the tonic and antiseptic mixture of Huxam's tinct., tinct. nux vom. and chlorate potash, but the first dose, at 11 a. m., was rejected. Having completed five days, without any serious developments, is good.

March 16th. The sixth day since the laparotomy in the case of Mrs. H. having presented no grave complications, I trust that nothing may occur in the future to interfere with her recovery. The temperature is still 97 3-4 degrees, with a pulse of 112 beats, and the stomach retaining her tonic mixture and light nourishment. There is slight tympanitis, without any marked sensitiveness upon palpation over the abdomen, so that the distension is doubtless due to gaseous accumulation in the intestines. Unless there should be some backset I will not give a daily report further, and you will please preserve the notes, as they may be desirable for publication.

March 18th. As the condition of our patient underwent quite a change yesterday, I have thought best to advise you of it. The temperature went up to 100 degrees in the afternoon, with a pulse of 140 to the minute; but there was nothing in the local features which served as an explanation; and finding the tongue dry, she was put upon a combination of spirits turp., carb. amm., camphor water and mucilage in the morning, which, however, was rejected after a few doses. I substituted turpentine capsules, and gave 10 grains of bisulphate quinine at night and 5 grains this morning. At 11 a. m. to-day the temperature has gone back to the old record, 97 3-4 degs., and pulse 130 beats. I have ordered Hoff's extract of malt, with bovanine, to-day. She has not been able to take the milk punch for some days, but takes coffee with a large proportion of milk, and she has not rejected this.

She will continue the turpentine capsules every six hours, and quinine morning and evening. Her spirits are good, and she is not suffering pain, so that no opiates are requisite.

March 22d. Since my last report of Mrs. H.'s case there has been nothing of special note until to-day. But a very grave feature has occurred, attended with great prostration, leading to apprehensions of collapse. The stomach not having retained either medicine or nourishment, she has been stimulated by hypodermics of brandy, alternated with ether, and in consultation with Drs. Earnest and Roy this evening a hypodermic of morphia and atropia was used with enema of bisul. quinine and tinct. of digitalis afterwards, the latter to be repeated every three hours. There is tympanitis, with tenderness over the abdomen, temperature 100 degrees, and pulse too feeble to be counted. The bowels were moved three times during the morning, but none since.

March 23d. You would infer from my card of last night that the end was near at hand in the case of Mrs. H., and she died at 2 a. m. this morning.

I made an autopsy, showing suppurative peritonitis involving the small intestines, but a very peculiar exclusion of the large intestines, with liver and stomach shut off by a layer of plastic lymph. The womb was bathed in degenerated sero-purulent exudation, but not perceptibly enlarged or inflamed.

Could the exact state of things have been known, the abdomen would have been opened and antiseptic irrigation been used; but our hindsights are always better than our foresights in these matters.

